# Investigation of *gyrA* and *parC* mutations and the prevalence of plasmid-mediated quinolone resistance genes in *Klebsiella pneumoniae* clinical isolates

**DOI:** 10.1186/s12866-024-03383-5

**Published:** 2024-07-18

**Authors:** Sepideh Rezaei, Saeed Tajbakhsh, Behrouz Naeimi, Forough Yousefi

**Affiliations:** 1 Department of Microbiology and Parasitology, School of Medicine, Bushehr University of Medical Sciences, Bushehr, Iran; 2 Student Research Committee, Bushehr University of Medical Sciences, Bushehr, Iran

**Keywords:** Fluoroquinolones, *Klebsiella pneumoniae*, Mutation, *gyrA*, *parC*, PMQR

## Abstract

**Background:**

The emergence of fluoroquinolone resistance in clinical isolates of *Klebsiella pneumoniae* is a growing concern. To investigate the mechanisms behind this resistance, we studied a total of 215 *K. pneumoniae* isolates from hospitals in Bushehr province, Iran, collected between 2017 and 2019. Antimicrobial susceptibility test for fluoroquinolones was determined. The presence of plasmid mediated quinolone resistance (PMQR) and mutations in quinolone resistance-determining region (QRDR) of *gyrA* and *parC* genes in ciprofloxacin-resistant *K. pneumoniae* isolates were identified by PCR and sequencing.

**Results:**

Out of 215 *K. pneumoniae* isolates, 40 were resistant to ciprofloxacin as determined by E-test method. PCR analysis revealed that among these ciprofloxacin-resistant isolates, 13 (32.5%), 7 (17.5%), 40 (100%), and 25 (62.5%) isolates harbored *qnrB*, *qnrS*, *oqxA* and *aac(6’)-Ib-cr* genes, respectively. Mutation analysis of *gyrA* and *parC* genes showed that 35 (87.5%) and 34 (85%) of the ciprofloxacin-resistant isolates had mutations in these genes, respectively. The most frequent mutations were observed in codon 83 of *gyrA* and codon 80 of *parC* gene. Single *gyrA* substitution, Ser83→ Ile and Asp87→Gly, and double substitutions, Ser83→Phe plus Asp87→Ala, Ser83→Tyr plus Asp87→Ala, Ser83→Ile plus Asp87→Tyr, Ser83→Phe plus Asp87→Asn and Ser83→Ile plus Asp87→Gly were detected. In addition, Ser80→Ile and Glu84→Lys single substitution were found in *parC* gene.

**Conclusions:**

Our results indicated that 90% of isolates have at least one mutation in QRDR of *gyrA* or*parC* genes, thus the frequency of mutations was very significant and alarming in our region.

## Background

Fluoroquinolones (FQs) are commonly used as effective antibiotics for the treatment of most infections caused by Gram-negative bacteria [1]. Unfortunately, due to their extensive use, FQ resistance (FQ-R) is on the rise in clinically important bacteria, including *Klebsiella pneumoniae* [1, 2]. FQs inhibit the activity of DNA gyrase and topoisomerase IV enzymes, which are essential enzymes for bacterial DNA replication and survival [2]. The main mechanisms of resistance to FQs in *Enterobacteriaceae* arise from chromosomal mutations in the quinolone resistance-determining region (QRDR), particularly of *gyrA* and *parC* encoding DNA gyrase, and topoisomerase IV, respectively. These mutations lead to structural changes in DNA gyrase and/or topoisomerase IV, which impairs the affinity to FQs [3]. Plasmid-mediated quinolone resistance (PMQR) genes are alternative mechanism of quinolone resistance. However, PMQR has been shown to emerge even in the absence of FQ therapy [4]. Three types of PMQR determinants have been identified in *Enterobacteriaceae*: (i) Qnr proteins, encoded by *qnr* genes (*qnrA*, *qnrB*, *qnrC*, *qnrD* and *qnrS*), belong to the pentapeptide repeat family and protect DNA gyrase and topoisomerase IV from quinolone inhibition by binding to them [1, 5]. (ii) The AAC(6’)-Ib-cr enzyme, a variant aminoglycoside acetyltransferase encoded by *aac(6’)-Ib-cr* gene, can modify ciprofloxacin with a piperazinyl substituent, reducing its activity [6]. (iii) OqxAB and QepA pumps reduce susceptibility to FQs by drug extrusion from the cell. These multidrug efflux pumps belong to the resistance–nodulation–cell division (RND) family and the major facilitator superfamily (MFS), respectively [2, 7]. The patterns of resistance mechanisms to FQ vary across different countries due to geographical impact on the emergence and dissemination of FQ-R mechanisms [1]. Thus, it is important to determine the major FQ-R mechanisms in each geographical area. The aim of this study was to determine chromosomal mutations in *gyrA* and *parC*genes as well as the prevalence of PMQR genes among fluoroquinolone resistant *K. pneumoniae* isolates in Bushehr province, Iran.

## Methods

### Bacterial isolation and identification

This project was approved by the Ethical Committee of Bushehr University of Medical Sciences with reference number IR.BPUMS.REC.1400.133. A total of 215 *K. pneumoniae* isolates were collected from six hospitals located in Bushehr province, in the south of Iran from November 2017 to February 2019. Bacterial identification was conducted by biochemical tests and confirmed by PCR to target malate dehydrogenase (*mdh*), the genus-specific housekeeping gene (Table 1) [8–10].

**Table 1:** Oligonucleotide primers used for detection PMQR determinants and mutation in QRDR

Target gene	Primer Sequence (5’-3’)	Annealing temp. (^0^C)	Amplicon size (bp)	Reference
*mdh*	F: GCGTGGCGGTAGATCTAAGTCATA R: TTCAGCTCCGCCACAAAGGTA	55	364	[8]
*qnrA*	F: ATTTCTCACGCCAGGATTTG R: GATCGGCAAAGGTTAGGTCA	56	516	[11]
*qnrB*	F: GATCGTGAAAGCCAGAAAGG R: ACGATGCCTGGTAGTTGTCC	57	469	[12]
*qnrC*	F: GGGTTGTACATTTATTGAATC R: TCCACTTTACGAGGTTCT	55	447	[13]
*qnrD*	F: CGAGATCAATTTACGGGGAATA R: AACAAGCTGAAGCGCCTG	56	582	[13]
*qnrS*	F: ACGACATTCGTCAACTGCAA R: TAAATTGGCACCCTGTAGGC	56	417	[12]
*aac(6’)-Ib-cr*	F: TTGGAAGCGGGGACGGAM R: ACACGGCTGGACCATA	55	265	[14]
*oqxA*	F: CCGCACCGATAAATTAGTCC R: GGCGAGGTTTTGATAGTGGA	55	313	[15]
*qepA*	F: CTGCAGGTACTGCGTCATG R: CGTGTTGCTGGAGTTCTTC	57	403	[16]
*gyrA*	F: CGCGTACTATACGCCATGAACGTA R: ACCGTTGATCACTTCGGTCAGG	60	441	[17]
*parC*	F: CAGCTCGGCATACTTCGAC R: CCTGAACTACTCCATGTACGTGAT	59	340	[4]

### Antimicrobial susceptibility testing

Antimicrobial susceptibility testing was determined by disk diffusion method for ciprofloxacin (5 mg) and levofloxacin (5 mg) in accordance with the Clinical and Laboratory Standards Institute (CLSI) guidelines [18]. In addition, MIC of ciprofloxacin was determined using E-tests (Liofilchem, Italy) on Mueller-Hinton agar (Biolife, Italy). *Escherichia coli* ATCC 25922 was used as the control strain for the antibiotic susceptibility tests.

### Detection of plasmid-mediated quinolone resistance genes

Plasmid-mediated quinolone resistance genes, including *qnrA*, *qnrB*, *qnrC*, *qnrD*, *qnrS*, *aac(6’)-Ib-cr*, *oqxA*, and *qepA* in ciprofloxacin-resistant isolates, were detected by PCR and sequencing performed by Bioneer Company (South Korea). The primers and PCR conditions used in this study were listed in Table 1.

### Detection of mutations in QRDR of *gyrA* and *parC* genes

A total of 40 ciprofloxacin-resistant isolates were selected. PCR amplification of *gyrA* and *parC* genes were carried out using primers and conditions listed in Table 1. A total reaction volume of 25 µl contained 12.5 µl 2x MasterMix (Ampliqon, Odense, Denmark), 1 µl (10 µmol) of each forward and reverse primer, 1 µl of template DNA; and 9.5 µl of nuclease-free water. The amplification conditions were as follows: pre-denaturation at 95 °C for 5 min; 30 cycles of denaturation at 94 °C for 1 min, annealing at 60 °C/59°C (*gyrA* / *parC*) for 30 s, and extension at 72 °C for 30 s, followed by a final extension at 72 °C for 5 min. Nucleotide sequencing of the PCR products was performed by the Bioneer Company (Seoul, Korea). Mutations in *gyrA* and *parC* genes for the 40 ciprofloxacin-resistant *K. pneumoniae* isolates were compared with the reference sequences of *gyrA* gene of *K. pneumoniae* ATCC13883 (GenBank accession number: DQ673325) and *parC* gene of *K. pneumoniae* ATCC1388T (GenBank accession number: AF303641). Online sequence alignment and analysis were performed using the online ClustalW2 multiple sequence alignment program.

### Nucleotide sequence accession number

The sequences of detected genes were submitted to the GenBank database under accession numbers OQ281591 - OQ281600.

## Results

### FQ susceptibility

Antimicrobial susceptibility testing (AST) revealed that among 215 of *K. pneumoniae* clinical isolates, 49 isolates (22.7%) were resistant to ciprofloxacin disk, 15 isolates (7%) were intermediate, and 151 isolates (70.2%) were susceptible in disk diffusion method. Moreover, among 64 isolates not susceptible to ciprofloxacin, 31, 7 and 26 isolates were resistant, intermediate, and susceptible to levofloxacin disk, respectively. In addition, out of 49 ciprofloxacin-resistant isolates, 40 isolates were resistant by E-test strips (MIC ≥ 4 µg/ml). These 40 ciprofloxacin-resistant isolates were selected for detection of PMQR genes and mutation in QRDR of *gyrA* and *parC* genes (Table 2).

**Table 2:** Antimicrobial susceptibility testing of quinolones, PMQR, and mutations in QRDR of *gyrA* and *parC* genes of 40 *K. pneumoniae* isolates

ID	Source	Antimicrobial susceptibility testing		PMQR	*qnrA*	*qnrB*	*qnrC*	*qnrD*	*qnrS*	*acc (6’) Ib-cr*	*qepA*	*oqxA*	QRDR	*gyrA*		*parC*	
		CIP	LEV											**Ser83**	**Asp87**	**Ser80**	**Glu84**
Kp5	Urine	≥ 32	R	2	-	-	-	-	-	+	-	+	2	Ile83*	WT	Ile80*	WT
Kp6	Urine	≥ 32	R	2	-	-	-	-	-	+	-	+	3	Phe83*	Ala87*	Ile80*	WT
Kp9	Urine	≥ 32	S	1	-	-	-	-	-	-	-	+	0	WT	WT	WT	WT
Kp11	Urine	≥ 32	R	2	-	-	-	-	-	+	-	+	3	Tyr83*	Ala87*	Ile80*	WT
Kp12	Feces	4	R	2	-	+	-	-	-	-	-	+	0	WT	WT	WT	WT
Kp21	Urine	≥ 32	R	2	-	-	-	-	-	+	-	+	3	Tyr83*	Ala87*	Ile80*	WT
Kp28	Urine	≥ 32	I	2	-	-	-	-	-	+	-	+	3	Phe83*	Ala87*	Ile80*	WT
Kp30	Burn	≥ 32	R	2	-	-	-	-	+	-	-	+	2	Ile83*	WT	Ile80*	WT
Kp31	Burn	≥ 32	R	2	-	-	-	-	+	-	-	+	2	Ile83*	WT	Ile80*	WT
Kp32	Burn	≥ 32	R	2	-	-	-	-	+	-	-	+	2	Ile83*	WT	Ile80*	WT
Kp43	Urine	≥ 32	R	2	-	-	-	-	-	-	-	+	3	Ile83*	Tyr87*	Ile80*	WT
Kp65	${\text{ET}}\mathop {\text{T}}\limits^{\text{a}}$	≥ 32	R	3	-	+	-	-	-	+	-	+	2	Ile83*	WT	Ile80*	WT
Kp69	Urine	≥ 32	R	3	-	-	-	-	+	+	-	+	3	Phe83*	Ala87*	Ile80*	WT
Kp71	Urine	≥ 32	R	2	-	-	-	-	-	+	-	+	3	Phe83*	Asn87*	WT	Lys84*
Kp81	Urine	≥ 32	R	3	-	+	-	-	-	+	-	+	2	Ile83*	WT	Ile80*	WT
Kp89	Blood	≥ 32	R	3	-	+	-	-	-	+	-	+	2	Ile83*	WT	Ile80*	WT
Kp92	Wound	≥ 32	R	2	-	-	-	-	-	+	-	+	2	Ile83*	WT	Ile80*	WT
Kp94	Blood	≥ 32	R	2	-	-	-	-	-	+	-	+	2	Ile83*	WT	Ile80*	WT
Kp96	ETT	≥ 32	R	2	-	-	-	-	-	+	-	+	3	Phe83*	Ala87*	Ile80*	WT
Kp97	Urine	≥ 32	R	2	-	-	-	-	-	+	-	+	2	Ile83*	WT	Ile80*	WT
Kp101	Blood	≥ 32	R	2	-	-	-	-	-	+	-	+	3	Phe83*	Ala87*	Ile80*	WT
Kp102	ETT	≥ 32	R	3	-	+	-	-	-	+	-	+	2	Ile83*	WT	Ile80*	WT
Kp106	Blood	≥ 32	R	2	-	-	-	-	-	+	-	+	2	Ile83*	WT	Ile80*	WT
Kp109	Urine	12	R	1	-	-	-	-	-	-	-	+	3	Ile83*	Gly87*	Ile80*	WT
Kp110	Urine	12	S	2	-	+	-	-	-	-	-	+	0	WT	WT	WT	WT
Kp121	Wound	≥ 32	R	2	-	-	-	-	-	+	-	+	3	Phe83*	Asn87*	WT	Lys84*
Kp128	Urine	≥ 32	R	3	-	+	-	-	-	+	-	+	2	Ile83*	WT	Ile80*	WT
Kp144	Urine	≥ 32	R	1	-	-	-	-	-	-	-	+	2	Ile83*	WT	Ile80*	WT
Kp147	Urine	≥ 32	R	2	-	-	-	-	-	+	-	+	3	Tyr83*	Ala87*	Ile80*	WT
Kp150	Urine	≥ 32	R	3	-	-	-	-	+	+	-	+	2	Ile83*	WT	Ile80*	WT
Kp155	Urine	≥ 32	R	3	-	+	-	-	-	-	-	+	3	Phe83*	Ala87*	Ile80*	WT
Kp162	Urine	≥ 32	R	3	-	-	-	-	+	+	-	+	2	Ile83*	WT	Ile80*	WT
Kp164	Urine	≥ 32	S	1	-	-	-	-	-	-	-	+	1	WT	WT	Ile80*	WT
Kp181	ETT	12	R	2	-	+	-	-	-	-	-	+	2	Ile83*	WT	Ile80*	WT
Kp184	Urine	≥ 32	R	3	-	+	-	-	-	+	-	+	2	Ile83*	WT	Ile80*	WT
Kp188	Urine	4	S	1	-	-	-	-	-	-	-	+	1	WT	Gly87*	WT	WT
Kp190	Urine	8	R	3	-	+	-	-	-	+	-	+	3	Phe83*	Ala87*	Ile80*	WT
Kp191	Urine	4	I	3	-	+	-	-	-	+	-	+	1	WT	Gly87*	WT	WT
Kp200	Urine	8	R	2	-	+	-	-	-	-	-	+	2	Ile83*	WT	Ile80*	WT
Kp206	Urine	4	R	2	-	-	-	-	+	-	-	+	0	WT	WT	WT	WT

ETT: Endotracheal tube secretions; CIP: ciprofloxacin; LEV: levofloxacin; QRDR: quinolone resistance-determining regions; PMQR: plasmid-mediated quinolone resistance; WT: wild type

### Characterization of PMQR genes

Molecular analysis revealed all 40 (100%) ciprofloxacin-resistant isolates carried at least one PMQR determinant: 40 (100%), 13 (32.5%), 7 (17.5%), and 25 (62.5%) isolates harbored *oqxA, qnrB*, *qnrS*, and *aac(6’)-Ib-cr* genes, respectively. Therefore, the prevalence of PMQR genes in our ciprofloxacin-resistant *K. pneumoniae* clinical isolates was 100%. In this study *qepA*, *qnrC*, *qnrD*, and *qnrA* genes were not found. Notably, as shown in Table 2, coexistence of 2 and 3 PMQR genes was found in 23 (57.5%) and 12 (30%) ciprofloxacin-resistant isolates, respectively. The remaining 5 isolates only carried *oqxA* gene.

### Mutations in QRDR of *gyrA* and *parC* genes

Analysis of mutations in *gyrA* and *parC* genes indicated that out of 40 ciprofloxacin-resistant *K. pneumoniae* isolates, 35 (87.5%) and 34 isolates (85%) had mutations in *gyrA* and *parC* genes, respectively. The most mutations were observed in Ser 83 of *gyrA* and Ser 80 of *parC* gene (Table 2). As shown in Table 3, single *gyrA* substitution including Ser83→ Ile and Asp87→Gly, and double *gyrA* substitutions including Ser83→Phe plus Asp87→Ala, Ser83→Tyr plus Asp87→Ala, Ser83→Ile plus Asp87→Tyr, Ser83→Phe plus Asp87→Asn, and Ser83→Ile plus Asp87→Gly were detected. The most common mutation in *gyrA* gene was Ser83→ Ile, which was present in 21 (52.5%) isolates. In addition, 14 isolates (35%) had double mutations in the *gyrA* gene (Table 3). Mutation analysis of *parC* gene indicated that the most common mutation in *parC* gene was Ser80→Ile, which was detected in 32 (80%) isolates. Moreover, 2 (5%) isolates had Glu84→Lys amino acid substitution in *parC* gene (Table 3).

**Table 3:** The frequency of mutations and amino acid changes within *gyrA*, and *parC* of 40 ciprofloxacin –resistant isolates

Gene	Amino acid position	Nucleotide changes	Amino acids substitute	No. of isolates (%)
***gyrA***	Serine 83	TCC→ATC	Isoleucine	19 (47.5%)
	Serine 83/	TCC→TTC	Phenylalanine	7 (17.5%)
	Aspartate 87	GAC→ GCC	Alanine	
	Serine 83/	GAC→ TAC	Tyrosine	3 (7.5%)
	Aspartate 87	GAC→GCC	Alanine	
	Serine 83/	GAC→AAC	Isoleucine	1 (2.5%)
	Aspartate 87	GAC→TAC	Tyrosine	
	Serine 83/	TCC→TTC -	Phenylalanine	2 (5%)
	Aspartate 87	GAC→AAC	Asparagine	
	Serine 83/	TCC→ATC	Isoleucine	1 (2.5%)
	Aspartate 87	GAC→GGC	Glycine	
	Aspartate 87	GAC→GGC	Glycine	2 (5%)
	WT	-	-	5 (12.5%)
***parC***	Serine 80	AGC→ATT	Isoleucine	32 (80%)
	Glutamate 84	GAA→AAA	Lysine	2 (5%)
	WT	-	-	6 (15%)

WT: wild type

### Correlation of ciprofloxacin MIC values with mutations in the QRDRs of *gyrA* and *parC* genes

Notably, a significant correlation between the frequency of mutations in QRDR of *gyrA* and *parC* and ciprofloxacin MIC values was observed in the present study, as the results showed that 29 out of 31 isolates in which the MIC of ciprofloxacin was ≥ 32 µg/ml had 2 or 3 mutations in both *gyrA* and *parC* genes simultaneously, while in 4 resistant isolates in which the MIC of ciprofloxacin was equal to 4 µg/ml, two isolates had no mutations and the other two isolates had only one mutation in the *gyrA* gene. It is worth noting that there was no correlation between the MIC values of ciprofloxacin and the number of harbored PMQR genes.

## Discussion

Fluoroquinolones, such as ciprofloxacin, are a group of effective drugs for the treatment of *Klebsiella pneumoniae* infections, which inhibit bacterial DNA replication by binding to topoisomerase IV and DNA gyrase enzymes [19]. Unfortunately, resistance to these antimicrobial agents has emerged, and the level of resistance is increasing due to their widespread use.

The main mechanism of resistance to FQs in bacteria is spontaneous mutations in the QRDR of *gyrA* gene encoding DNA gyrase and *parC* gene encoding topoisomerase IV, particularly at the highly conserved residues Ser83 and Asp87 of *gyrA* gene [20].

In the present study, we investigated mutations in the QRDR of *gyrA* and *parC* genes and the prevalence of PMQR genes among fluoroquinolone resistant *K. pneumoniae* clinical isolates.

Our results demonstrated that 18.6% of isolates were resistant to ciprofloxacin, which was in agreement with previous studies conducted by Jomehzadeh et al., (18.5%) [21], Razavi et al. (19.6%) [22], and Priyadarshini et al., (19.1%) [23]. In addition, 31 (77.5%) out of 40 resistant isolates showed high-level resistance (≥ 32 µg/ml) to ciprofloxacin. Different frequencies of high-level resistance were reported by Ghane et al., (18.5%), Esmaeel et al., (57.8%) [24], and Geetha et al., (88%) [25].

Although mutations in QRDR of *gyrA* and *parC* genes are the main cause of FQs resistance, PMQR genes contribute to fluoroquinolone resistance due to their high horizontal transferability [21].

Our molecular analysis identified that 40 (100%) ciprofloxacin-resistant isolates harbored at least one PMQR determinant and the most common PMQR gene among our isolates was *oqxA* gene (100%). Prevalence of PMQR genes in the study conducted by Jomehzadeh et al. [21] was 88% and *aac(6’)-Ib-cr* was detected as the most common PMQR gene (50%), while in another study done by Sani et al. 85.4% of the isolates harbored PMQR genes and the most prevalent PMQR gene was *qnrS* (41.67%) [26]. Furthermore, *qnrA* and *qepA* genes have not been reported in several studies conducted in some geographic areas, including Korea, Malaysia, and Iran which support our data for these genes [27, 28].

The main mechanism of resistance to FQ in the *Enterobacteriaceae* is alterations in QRDR of *gyrA*, which encodes DNA gyrase, a type II topoisomerase [29].

Mutation analysis in QRDR of *gyrA* gene revealed that 35(87.5%) out of 40 ciprofloxacin-resistant isolates had at least one mutation in *gyrA* gene and the most frequent amino acid substitution in *gyrA* gene was Ser83→Ile, which was found in 52.5% of our isolates. This finding was inconsistent with the studies done by Fu et al. (all ciprofloxacin-resistant isolates had Ser83→Leu or Ser83→ Ile substitutions) [29], and Azargun et al. (all ciprofloxacin-resistant isolates had Ser83→Leu substitution) [2]. However, it should be mentioned that in the present study among 6 isolates with low-level resistance to ciprofloxacin (MIC = 4–8 µg/ml) only one isolate had Ser83→Ile substitution. In addition, single substitution Ser83→Ile was detected in 19 (47.5%) ciprofloxacin-resistant isolates and 14 (35%) isolates had five types of double mutations at Ser83 and Asp87 positions. Fu et al. reported, among Ser83→Leu, Ser83→Ile, Ser83→Tyr, and Ser83→Phe single substitutions, only Ser83→Ile single substitution showed significantly different distribution between the ciprofloxacin-resistant and ciprofloxacin-susceptible isolates [29].

Additionally, Fu et al. reported out of eight types of double mutations involving both Ser83 and Asp87 only three double mutations including Ser83→Leu plus Asp87→Asn, Ser83→Phe plus Asp87→Asn, and Ser83→Tyr plus Asp87→Asn were associated with ciprofloxacin resistance [29]. In the study conducted by Azargun et al., Ser83→Leu plus Asp87→Asn double mutations were detected in 60% of FQ-resistant *K. pneumoniae* isolates [2]. In agreement with their findings, one of the five double mutations identified in our study was Ser83→Phe plus Asp87→Asn but the four remaining double mutations of the present study were not found in the Fu, and Azargun’s studies. Therefore, the effect of the other double mutations was ignored in the mentioned studies. It is notable that, in agreement with the present study, the most common double mutation observed in the Akya’s study was Ser83→Phe plus Asp87→Ala, which was present in 42.9% of FQ-resistant *K. pneumoniae* isolates [27]. Moreover, similar to the current study Ser83→Phe plus Asp87→Asn double mutations were detected in 14.3% of FQ-resistant *K. pneumoniae* isolates. However, the three remaining double mutations of the present study were not found in their study.

Although amino acid substitutions at other positions of *gyrA* QRDR including Glu94, Arg154, Thr161, Ala171, Gly177and Leu 187 were reported [27, 29], Anuar et al., indicated ciprofloxacin resistance was significantly associated with *gyrA* alteration in Ser83 (*p* = 0.003), Asp87 (*p* = 0.005) or both of them (*p* = 0.016) [30]. Supporting this finding in the present study, amino acid substitutions in QRDR of *gyrA* gene at positions other than Ser83 and Asp87 were not found. Another factor that affects resistance to FQ is mutation in the QRDR of *parC* gene. Mutation analysis of *parC* QRDR revealed that 34 (85%) isolates had mutations in *parC* gene including Ser80→Ile (80%) and Glu84→Lys (5%). In the studies done by Azargun et al., [2] 60%, and by Akya et al., [27] 53.6% of FQ-R *K. pneumoniae* isolates had Ser80→Ile amino acid substitutions. Moreover, in Akya’s study Glu84→Lys amino acid substitution was found in 25% of FQ-R *K. pneumoniae* isolates [27].

In addition, in our study 14 (35%) isolates had three mutations in QRDR of both *gyrA* and *parC* genes. Notably, the frequency of mutations in QRDR of *gyrA* and *parC* revealed a significant effect on ciprofloxacin MIC values; as the results showed 29 isolates out of 31 isolates in which the MIC of ciprofloxacin was ≥ 32 µg/ml had 2 or 3 mutations in both *gyrA* and *parC* genes simultaneously, while in 4 resistant isolates in which the MIC of ciprofloxacin was equal to 4 µg/ml, two isolates had no mutations and the other two isolates had only one mutation in the *gyrA* gene.

## Conclusion

In conclusion, all ciprofloxacin-resistant *K. pneumoniae *isolates either had mutations in the QRDR of *gyrA* and *parC* genes or carried PMQR genes.  Our results showed 90% of ciprofloxacin-resistant *K. pneumoniae* isolates had at least one mutation in QRDR of *gyrA* or*parC* genes, thus the frequency of mutation in QRDR was very significant and alarming in our region. Amino acid substitution Ser83→Ile in *gyrA* which has the greatest impact on ciprofloxacin resistance and Ser80→Ile in *parC* genes were the most frequent mutations among our FQ-R *K. pneumoniae* isolates. In addition, acquisition of 2 or 3 mutations in both *gyrA* and *parC* genes played an important role in conferring high level resistance to ciprofloxacin.

## Data Availability

The sequences of detected genes were submitted to the GenBank database under accession numbers OQ281591 - OQ281600.

## Abbreviations

PMQR: Plasmid mediated quinolone resistance
QRDR: Quinolone resistance-determining region
FQs: Fluoroquinolones
RND: Resistance–nodulation–cell division
MFS: Major facilitator superfamily
